# Bleeding due to successive duodenal and rectal ulcers in an 81-year-old patient with severe COVID-19: a case report

**DOI:** 10.1186/s12877-023-04283-5

**Published:** 2023-09-14

**Authors:** Guanlin Liu, Qiang Meng, Yunwei Li, Yiling Li, Taiwei Jiao, Hongwen Zhao, Bing Dai

**Affiliations:** 1 Department of Anorectal Surgery, The First Affiliated Hospital of China Medical University, Shenyang, China; 2 Department of Anorectal Surgery, The First Affiliated Hospital of China Medical University, No. 155, Nanjing North Street, Heping District, Shenyang, 110001 Liaoning China; 3 Department of Gastroenterology, The First Affiliated Hospital of China Medical University, Shenyang, China; 4 Department of Respiratory and Critical Care Medicine, The First Affiliated Hospital of China Medical University, Shenyang, China

**Keywords:** COVID-19, SARS-CoV-2, Case report, Gastrointestinal bleeding, Rectal ulcer, Duodenal ulcer

## Abstract

**Background:**

In the early stages of the coronavirus disease 2019 (COVID-19) outbreak, the most widely recognised symptoms of the disease were fever, cough, shortness of breath, myalgia, and fatigue. However, in addition to these symptoms, COVID-19 can cause systemic symptoms outside the lungs. Older patients with severe COVID-19 often require admission to the intensive care unit (ICU). Acute rectal ulcer bleeding, characterised by painless, profuse haematochezia, caused by solitary or multiple rectal ulcers, is one of the main causes of severe haematochezia in patients with COVID-19 in the ICU. However, recurrent duodenal ulcer bleeding followed by rectal ulcer bleeding has not previously been reported in older patients during ICU treatment for severe COVID-19.

**Cases presentation:**

Herein, we report the case of an 81-year-old woman admitted to the emergency department due to severe COVID-19 and transferred to the ICU 2 days later for treatment. During treatment in the ICU, the patient developed recurrent duodenal ulcer bleeding and underwent endoscopic electrocoagulation haemostasis and gastroduodenal artery embolisation. However, the night after the final haemostatic operation, due to rectal ulcer bleeding, the patient discharged bloody stools intermittently, which was effectively controlled using endoscopic electrocoagulation, topical medication, blood transfusion, and haemostatic drugs.

**Conclusions:**

To the best of our knowledge, this is the first report of duodenal ulcer bleeding followed by rectal ulcer bleeding in an older patient with severe COVID-19 infection. This report creates awareness for clinicians about the multiple and complex gastrointestinal symptoms that may occur during COVID-19 treatment.

## Background

Severe acute respiratory syndrome coronavirus (SARS-CoV)-2 is a novel coronavirus that causes coronavirus disease 2019 (COVID-19), which has been an epidemic since 2019 [1]. According to the World Health Organization, as of 21 March 2023, there were > 760.4 million confirmed cases of COVID-19 globally, resulting in > 6.87 million COVID-19-related deaths [2]. In the early stage of the COVID-19 outbreak, respiratory manifestations, myalgia, and fatigue were the most commonly reported symptoms. However, as the pandemic progressed and the number of affected patients increased, the associated clinical symptoms became more diverse and complex, and gastrointestinal symptoms, such as anorexia, vomiting, diarrhoea, and abdominal pain, are currently frequently observed clinical manifestations of COVID-19 [3]. Older individuals are more likely to be infected with COVID-19 for various reasons [4], and patients with severe COVID-19 often need to be admitted to the intensive care unit (ICU) for treatment. According to Lin Chengkuan, acute rectal ulcer bleeding is an important cause of severe haematochezia among patients in the ICU (occurring in approximately 1% of patients in the ICU) [5], and is characterised by painless and massive haematochezia caused by solitary or multiple rectal ulcers, usually ranging from 3–10 cm above the dentate line, for which the pathogenesis is currently unclear [6]. To the best of our knowledge, duodenal and rectal ulcer bleeding occurring successively during the treatment of severe COVID-19 in older adults has not previously been reported. Herein, we report the case of an older patient with severe COVID-19 who experienced recurrent duodenal and rectal ulcer bleeding during hospitalisation. The patient's bleeding-related symptoms were effectively controlled using a multiple-treatment approach. In this report, we systematically described the disease progression and treatment process of this case and, combined with recent relevant literature reports, preliminarily explored the causes of gastroduodenal and rectal ulcer bleeding in patients with SARS-CoV-2 infection.

## Case presentation

An 81-year-old female patient was transferred to the emergency centre of our hospital because of cough and persistent high fever and was subsequently diagnosed with severe COVID-19-related pneumonia. The patient had a medical history of spontaneous pneumothorax, hypertension, and a lumbar fracture but no history of diabetes, heart disease, or immunodeficiency-related diseases. On the day of admission, a nasopharyngeal swab tested positive for COVID-19 nucleic acid. Chest computed tomography examination revealed that the patient had multiple infectious lesions in both lungs, as well as pneumothorax in the right pleural effusion. The test results further revealed that the patient had hypoproteinaemia (albumin: 21.8 g/L) (Table 1).

**Table 1:** Test results during hospitalisation

**Days of admission, NO**	**1**	**3**	**5**	**7**	**9**	**11**	**13**	**15**	**17**	**18**	**19**	**21**	**23**	**25**	**26**	**27**	**29**	**31**	**33**	**35**	**37**	**39**
Results of novel coronavirus nucleic acid in nasopharyngeal swab	( +)	( +)	( +)	( +)	( +)	( +)	( +)	(-)	(-)	( +)	( +)	(-)	(-)	(-)	(-)	(-)	(-)	( +)	( +)	(-)	(-)	(-)
Haemoglobin, g/dL	12.5	9.3	10.1	8.8	10.1	9	8.8	10.1	9	10.2	13	8.8	9.1	9.5	9	11.4	10.6	9.7	9.3	9	10.7	9.3
White blood cell, 10^9^/L	7.47	7.57	5.46	8.22	8.55	12.94	10.68	5.26	3.49	6.42	9.74	4.28	3.3	3.16	1.9	1.91	6.48	3.26	0.86	1.8	4.94	4.11
Neutrophils, 10^9^/L	6.71	7.34	4.87	7.59	8.23	12.42	10.15	4.88	3.12	5.98	9.11	4.03	3.08	2.97	1.73	1.7	6.16	2.95	0.75	1.68	4.75	3.96
Lymphocyte, 10^9^/L	0.23	0.05	0.14	0.21	0.08	0.12	0.1	0.13	0.27	0.28	0.28	0.08	0.14	0.07	0.08	0.08	0.16	0.14	0.06	0.06	0.06	0.06
Granulocyte ratio, %	89.8	97	89.2	92.3	96.2	96	95	92.7	89.4	93.1	93.5	94.1	93.4	94.1	91.1	89	95.1	90.5	87.1	93.3	96.2	96.3
D-dimer, ug/mL	2.14	1.59	1.32	1.32	1.66	1.33	1.88	2	2.65	—	8.86	8.15	12.48	12.98	—	17.64	10.97	—	15.72	6.25	3.62	3
IL-6, pg/mL	—	—	—	7.66	4.99	31.9	62.55	249.2	263	1334	828.2	684	1710	1012	1004	> 5000	> 5000	> 5000	> 5000	3721	4526	2549
**Days of admission, NO**	**1**	**3**	**5**	**7**	**9**	**11**	**13**	**15**	**17**	**18**	**19**	**21**	**23**	**25**	**26**	**27**	**29**	**31**	**33**	**35**	**37**	**39**
C-reactive protein, mg/L	—	242.4	114.2	41	—	6.9	74.2	112.1	26.1	18.3	11	6.8	7.2	12	11.1	42.1	129.8	91.9	85.2	105.1	106	121.1
Procalcitonin, ng/mL	—	4.88	1.25	0.667	0.234	0.146	0.074	0.804	0.438	0.289	0.27	0.26	0.356	0.306	—	1.71	5.9	2.81	3.01	2.48	4.41	5.7
NT-proBNP, pg/mL	—	—	—	3137	1597	1587	1335	1544	809.5	918.3	1166	1399	727.2	—	—	4402	> 35,000	> 35,000	> 35,000	> 35,000	> 35,000	> 35,000
Sodium, mmol/L	137.4	139	141.6	143.7	141.1	141.2	149.4	143.8	140	136.7	143.3	141.7	140	142.6	143.8	140.3	145.7	143.7	144.1	143.9	145.8	151.9
Potassium, mmol/L	3.61	3.86	4.67	4.61	—	5.41	3.65	4.47	4.56	3.8	3.57	4.03	4.39	4.46	4.36	4.38	4.02	3.8	4.01	4.18	4.63	4.16
Urea, mmol/L	13.3	9.49	9.84	10	—	16.9	12.01	10.98	12.87	16.73	17.01	9.43	8.03	7.47	7.14	9.99	10.17	11.01	13.31	14.61	19.99	28.2
Cr, umol/L	84	48	41	45	—	41	37	35	49	57	54	46	46	33	42	53	60	52	47	38	38	44
Albumin, g/L	21.8	20.2	23.7	27.4	—	30.6	30.1	31.1	26.1	24.1	31	26.7	26.8	28.3	27.4	25.7	25.7	25.4	28.4	29.8	31.9	33.9
AST, U/L	—	26	15	11	—	19	14	13	17	28	33	63	97	51	42	45	53	54	111	71	57	48
ALT, U/L	23	19	14	10	—	15	11	12	14	14	22	45	65	42	41	43	35	32	50	56	57	56
LDH, U/L	—	—	—	204	—	—	—	—	260	—	369	334	390	—	—	399	424	454	477	535	583	667
**Days of admission, NO**	**1**	**3**	**5**	**7**	**9**	**11**	**13**	**15**	**17**	**18**	**19**	**21**	**23**	**25**	**26**	**27**	**29**	**31**	**33**	**35**	**37**	**39**
PT, s	14.7	17.2	14.5	14.2	12.4	13.8	15.1	14	14.8		17	15.1	15.5	15.5	15.7	15.4	17.7	16.4	15.7	21	21	21.9
APTT, s	35.2	39.1	42	31.9	27.2	25.2	32.2	31.7	30.3		32.2	38.8	46.6	43.1	50.8	47.7	59.5	53.9	48.5	61.2	50.2	54.4
Gastric occult blood	—	—	—	—	—	—	( +)	( +)	( +)	( +)	( +)	( +)	( +)	( +)	( +)	(+ -)	(+ -)	( +)	( +)	(-)	(-)	(-)
Faecal occult blood	—	—	—	—	—	—	—	( +)	( +)	( +)	( +)	( +)	( +)	( +)	( +)	( +)	( +)	( +)	(-)	(-)	(+ -)	(+ -)

*Abbreviations*: *IL-6* Interleukin-6, *NT-proBNP* N-terminal pro-brain natriuretic peptide, *Cr* Creatinine, *AST* Aspartate transaminase, *ALT* Alanine transaminase, *LDH* Lactate dehydrogenase, *PT* Prothrombin time, *APTT* Activated partial thromboplastin time

Based on consultation, during the emergency period, we performed preliminary medical treatment for patients with severe novel coronavirus-infection pneumonia and administered symptomatic treatment for other symptoms. These symptoms included type II respiratory failure, right-sided fluid pneumothorax, grade III hypertension, hypoproteinaemia, mild anaemia, and cardiac insufficiency. The treatment plan included high-flow humidified oxygen inhalation, antiviral, and anti-infective therapy (ertapenem + levofloxacin + paxlovid), airway expansion (methylprednisolone sodium succinate, doxofylline, and salbutamol the following day), gastric acid inhibition (roxatidine acetate hydrochloride), expectoration (ambroxol hydrochloride and acetylcysteine the following day), and albumin supplementation. The patient had difficulty urinating due to a lumbar fracture; therefore, she was provided with an indwelling catheter and nutritional support (18 kinds of amino acids, fat-soluble vitamins, water-soluble vitamins, and medium- and long-chain fat emulsions). After 2 days of emergency treatment, the patient was transferred to the ICU for further treatment.

On the 5th day of admission, we administered heparin sodium (12,500 U) to prevent thrombosis and conducted oral endotracheal intubation and ventilator-assisted ventilation at night. The next day (the 6th day of admission), she began receiving nasal feeding and enteral nutrition via a nasojejunal tube. On the 10th day after admission, 80 mL of dark brown gastric content was extracted via a gastric tube. Gastroduodenal bleeding was suspected; therefore, we administered somatostatin (3 mg) and omeprazole (80 mg) based on the original treatment plan, suspended the use of enteral nutrition, and advised the patient to abstain from drinking water and undergo gastrointestinal decompression therapy. On the 14th day of admission, the patient experienced diarrhoea thrice, and discharged black stools. We tested for gastric content occult blood, faecal occult blood, *Clostridium difficile*, and bacillus-to-coccus ratio in the stool of the patient, and the results were as follows: gastric content occult blood ( +), faecal occult blood ( +), and *Clostridium difficile* determination (-). The bacillus-to-coccus ratio in the stool increased (coccus: bacillus = 10:1); therefore, we increased the dosage of somatostatin (3 mg–6 mg), and administered vancomycin via nasal feeding. From the next day (the 15th day of admission), the patient was injected with octreotide subcutaneously (0.1 mg once every 8 h) and was orally administered bifid triple viable capsules (2 g once every 8 h) and combined *Bacillus subtilis* and *Enterococcus faecium* granules with multivitamins (250 mg once daily) for 3 consecutive days.

**Fig. 1:**
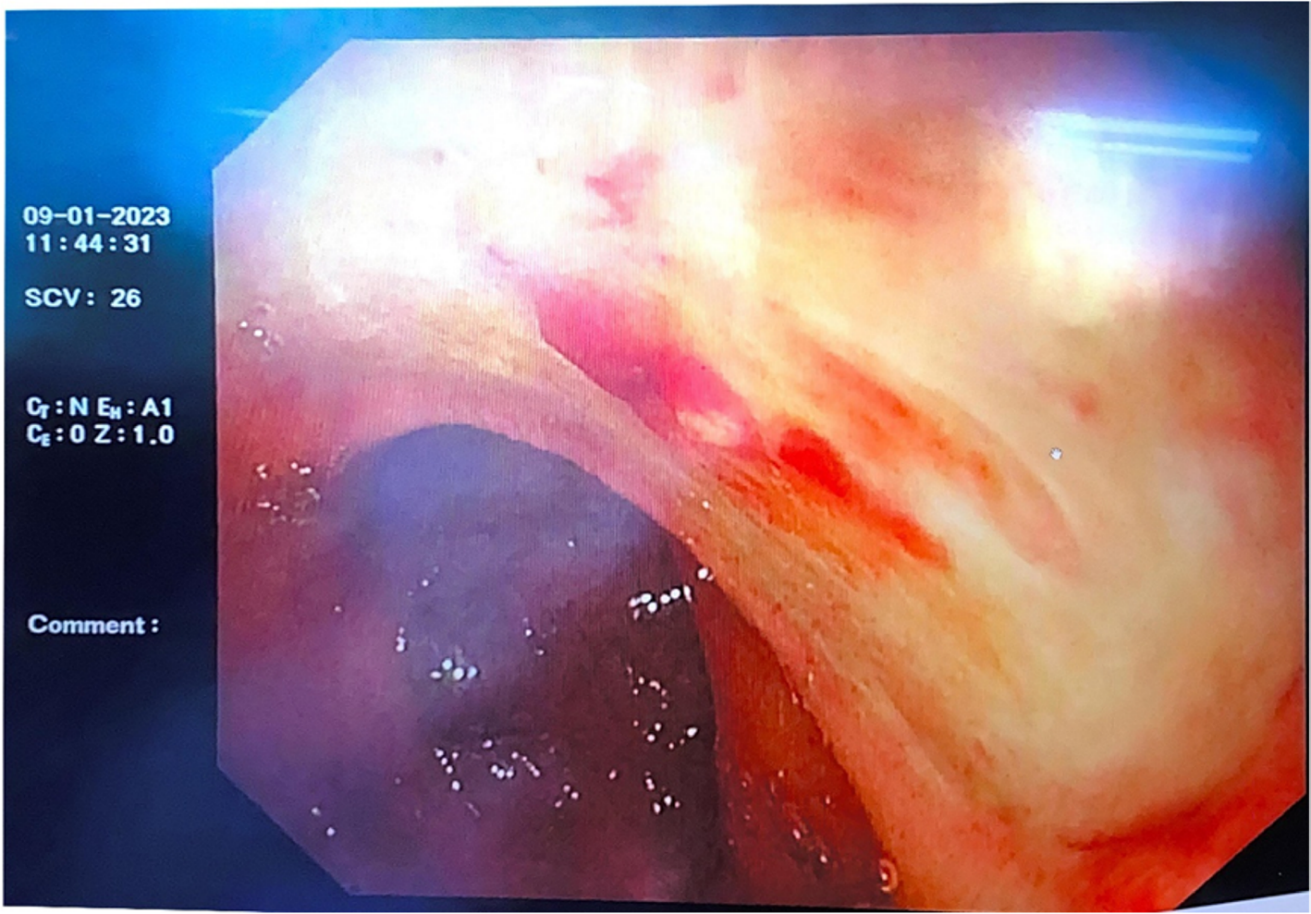
Ulcers in duodenal bulb showing post-electrocoagulation changes, with observable localised redness and blood crust adherence

**Fig. 2:**
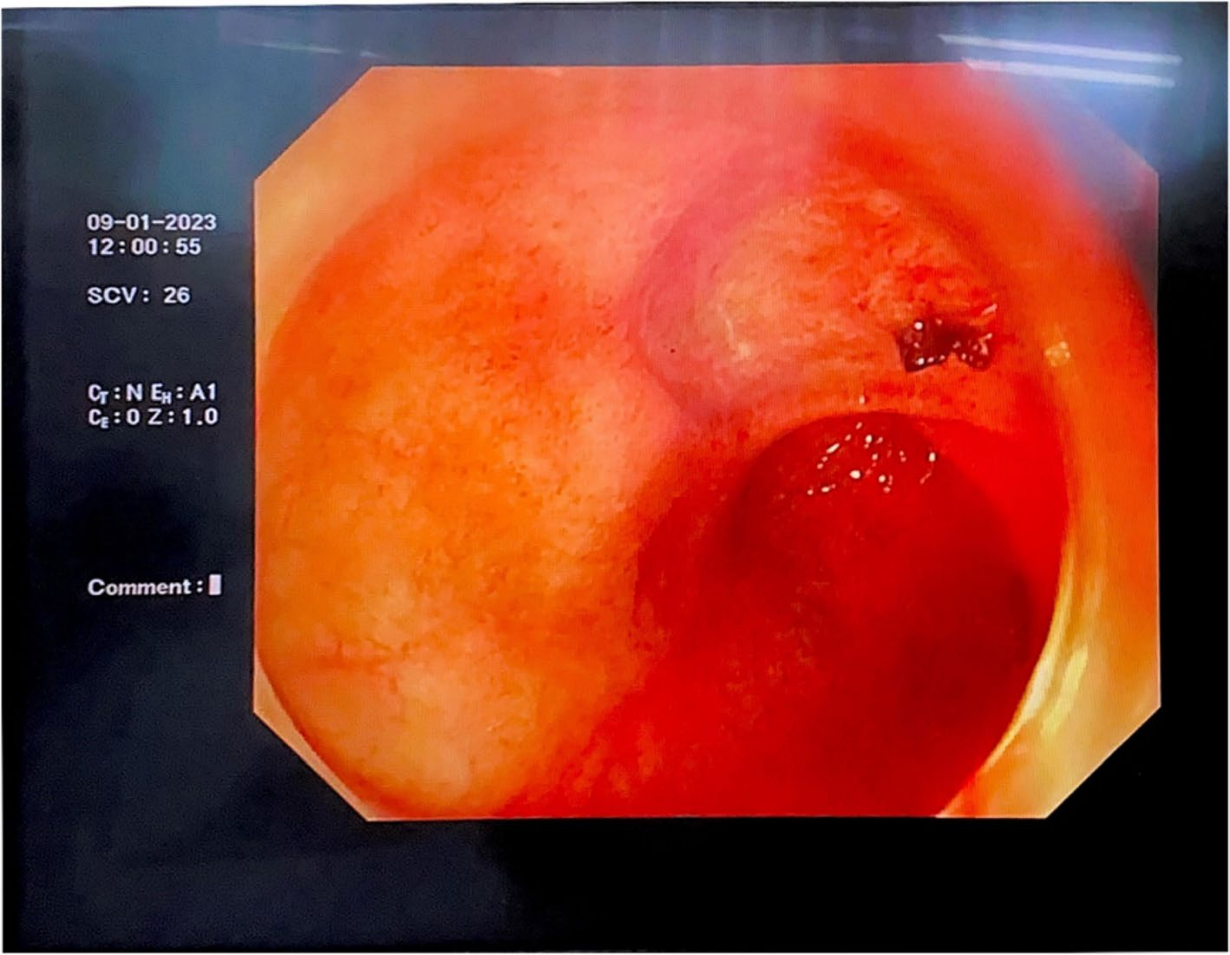
Haemostasis following electrocoagulation of the vascular stump using thermocoagulation forceps

However, 2 days later (the 17th day of admission), the patient suddenly vomited approximately 100 mL of fresh blood, and the laboratory test results showed mild anaemia, infection, inflammatory response, heart failure, and abnormal coagulation function (Table 1). An emergency gastroscopy was immediately performed. Patchy erosions were observed in the lower oesophagus and cardia. Fresh blood-filled gastric juice was observed in the gastric fundus. A 2.0-cm ulcer was observed on the anterior wall of the duodenal bulb on the minor curvature; the base was flat with white moss, and the surrounding mucosa was congested and oedematous. A vascular stump was observed at the base of the ulcer with pulsation and was considered to be the residual artery of the stomach. Therefore, we performed electrocoagulation haemostasis under endoscopy, and injected the patient with hemocoagulase agkistrodon (2 U) and somatostatin (6 mg) following the operation. The next day, the patient passed black stool; hence, active upper gastrointestinal tract bleeding was suspected. Therefore, we repeated an emergency gastroscopy, and the results revealed redness and a blood scab attached to the previous haemostatic ulcer; blood-filled intestinal fluid was visible in the descending duodenum, and pulsatile bleeding was observed after washing the vascular stump of the ulcer (Fig. 1). Under endoscopy, we used thermocoagulation forceps to pull up the residual blood vessels and applied electrocoagulation to halt the bleeding again (Fig. 2). After the operation, the patient was further administered parenteral hemocoagulase agkistrodon (2 U). This treatment controlled upper gastrointestinal bleeding. To prevent further bleeding, celiac arteriography and gastroduodenal artery embolisation were performed. Furthermore, we administered 6 mg of somatostatin and increased the dose of omeprazole (80–160 mg). However, the night after the operation, the patient discharged a small amount of bloody stool when she turned over, and her blood pressure gradually decreased. Dopamine and 2 U of filtered red blood cell suspension were administered urgently, and her blood pressure normalised.

On the 23rd day of admission, 50 mL of dark brown stomach contents were extracted again through the stomach tube, and a bedside gastroscopy was immediately performed. Patchy erosions were observed in the lower oesophagus and cardia. Erythema was observed in the gastric antrum; however, no ulcers or bleeding were observed. The base of the ulcer at the duodenal bulb, the previous bleeding site, had a flat, white, mossy appearance with congested and oedematous surrounding mucosa. The centre of the ulcer showed changes after electrocoagulation without residual blood vessels.

**Fig. 3:**
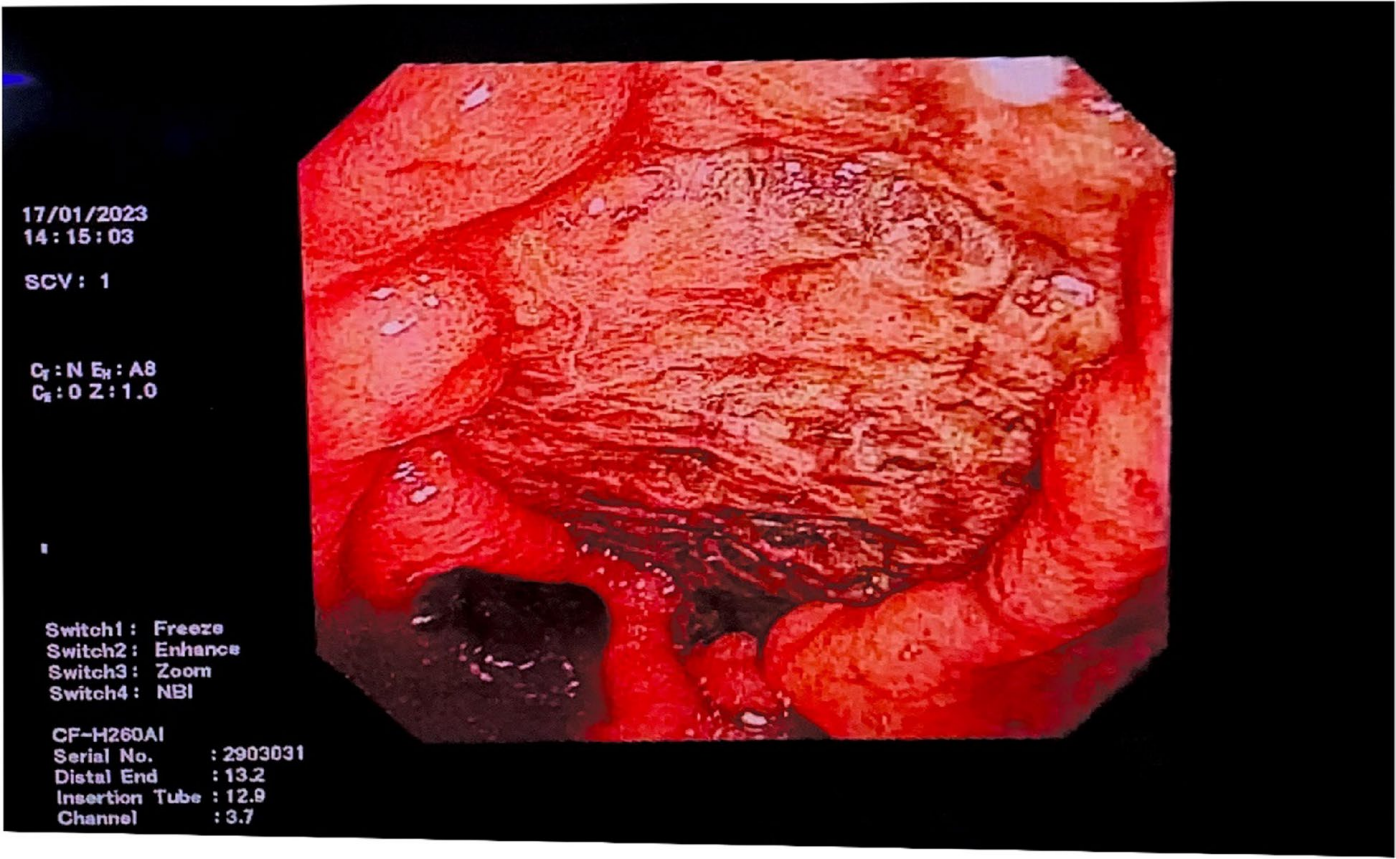
Rectal ulcer observed on e-colonoscopy prior to surgery (the 27th day after admission)

**Fig. 4:**
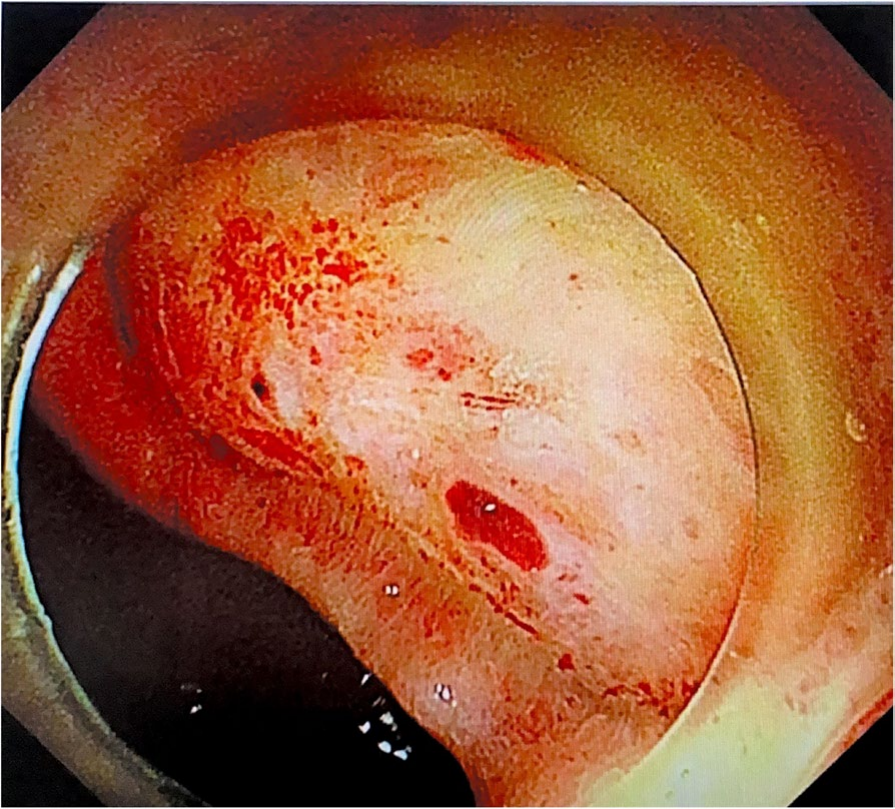
A 3.0-cm ulcer on the right anterior wall of the ampulla of the rectum. The anal side of the lesion is about 2.0 cm from the anal verge. The ulcer was regularly shaped and did not grow into the cavity. Pulsating blood vessel stumps on the side of the mouth can be observed

**Fig. 5:**
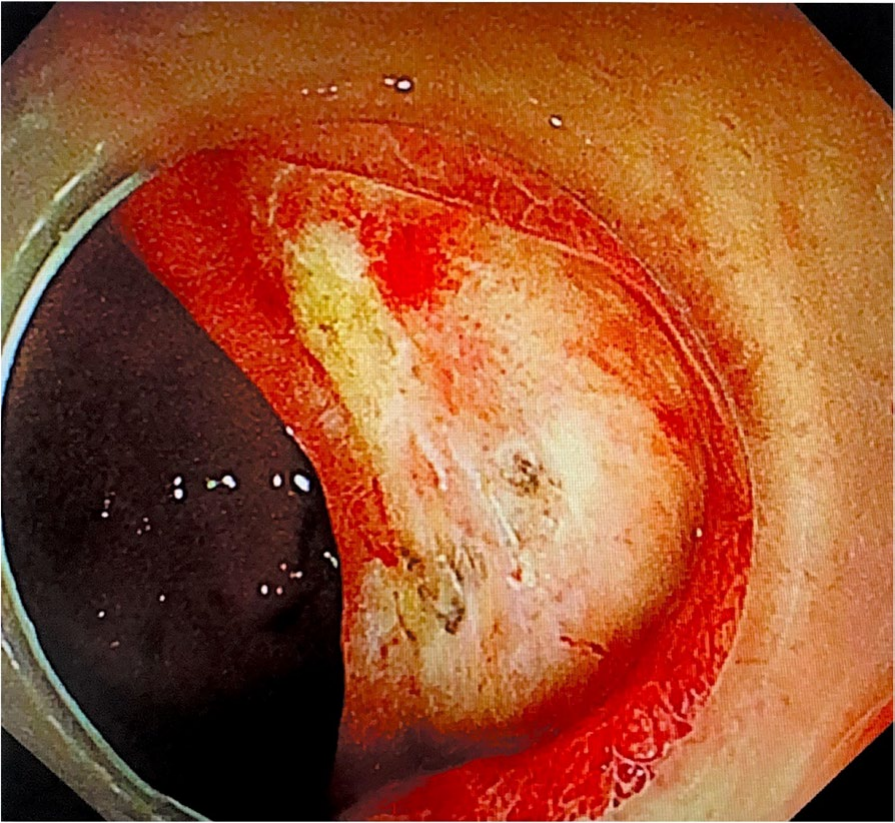
Bleeding from a rectal ulcer stopped after electrocoagulation

**Fig. 6:**
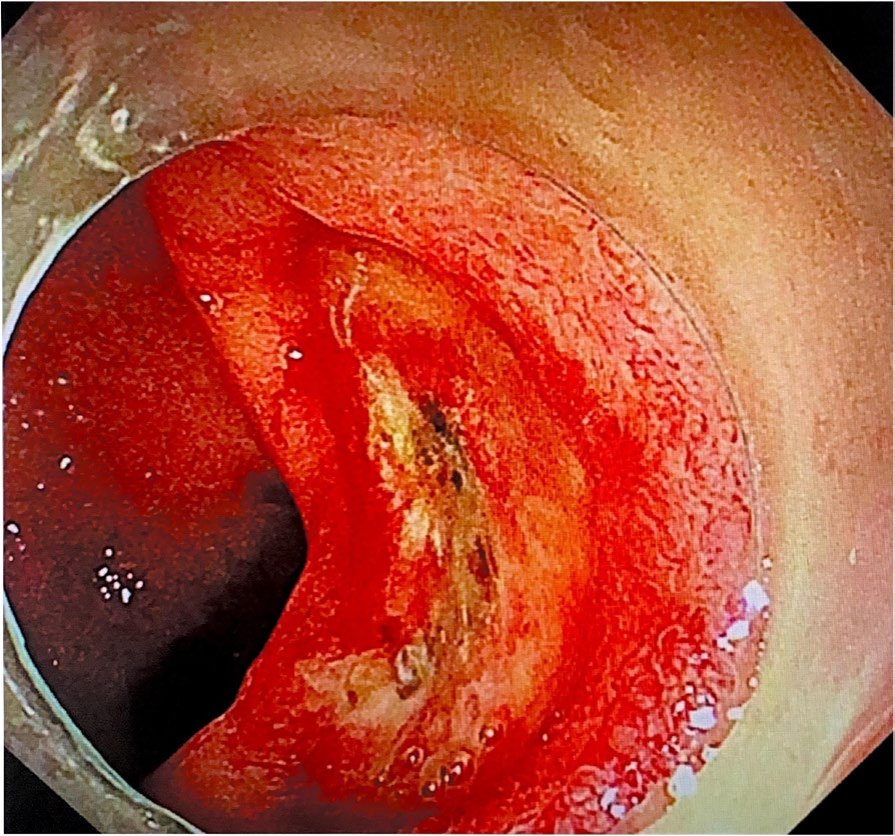
Bleeding from a rectal ulcer stopped after electrocoagulation

On the same night, the patient suddenly discharged 100 mL of fresh bloody stool. An infusion of 10 U leukocyte-filtered cryoprecipitate and two therapeutic doses of blood platelets collected using a leukofilter were immediately administered. However, the following night, she passed approximately 150 mL of bloody stool and continued to have blood in the stool for the next 2 days. During this period, she received 6 mg of somatostatin daily and 300 IU of the human prothrombin complex on the 25th day. On the 26th day of admission, the patient's haemoglobin count decreased compared to the previous day (Table 1), and we suspected that she had developed lower gastrointestinal bleeding. To determine the cause of the bloody stool, we performed a bedside colonoscopy, and found a 3.0-cm ulcer on the right anterior wall of the rectal ampulla. The lesion was approximately 2.0 cm from the anal margin. Pulsating vascular stumps were observed on the surfaces of the ulcers on the oral side (Figs. 3 and 4). After haemostasis using electrocoagulation forceps, the patient’s lower gastrointestinal bleeding ceased (Figs. 5 and 6). After the operation, she received 160 mg omeprazole and 6 mg somatostatin, and was infused with 1 g of fibrinogen. Simultaneously, human epidermal growth factor gel (twice daily) and antibacterial ointment containing chlorhexidine (once daily) were applied, and the patient was closely observed for bloody stool.

The next day (the 27th day of admission), the patient’s blood pressure progressively decreased, whereas her heart rate increased. However, even large vasopressor doses were difficult to maintain. The oxygen concentration in the ventilator was adjusted to 100%; however, the patient’s blood oxygen partial pressure was only 53 mmHg. Septic shock was considered based on the test results (Table 1). After a cardiothoracic surgical evaluation of the patient’s condition, extracorporeal membrane oxygenation (ECMO) was recommended. Subsequently, under the guidance of a cardiothoracic surgeon, the patient underwent ECMO through the right femoral vein and arterial catheterisation. Seven days later (the 33rd day of admission), the patient underwent a faecal occult blood test (immunological method), and the result was negative. Furthermore, her vital signs tended to be stable (Table 1). On the 41st day after admission, the ECMO membrane was replaced at the patient’s bedside. However, in the early morning and the morning of the following day (the 42nd day of admission), the patient excreted 250 mL of unformed black stool without any obvious cause. A bedside colonoscopy was performed to determine the cause of the black stool formation. During the procedure, the site of the previous colonoscopic haemostatic ulcer showed postoperative changes. The range significantly decreased, and only a small amount of blood oozed from the ulcer surface. We believe that this bleeding was caused by heparin present in the replaced ECMO membrane, which led to abnormal coagulation function in the patient. Therefore, no specific treatment was administered. The following day (the 43rd day after admission), the patient’s faecal occult blood test result was negative. Unfortunately, on the 66th day after admission, the patient died of multiple organ failure.

## Discussion and conclusions

SARS-CoV-2 is highly contagious and has caused a global pandemic in recent years. Patients with COVID-19 may exhibit symptoms, ranging from mild, flu-like symptoms to severe symptoms, such as acute respiratory distress syndrome, pneumonia, and multiple organ failure [7]. Age, sex, and the presence of underlying diseases are all relevant factors in determining disease severity. Older patients with COVID-19 are at a higher risk of severe complications and adverse outcomes, often requiring ICU treatment [8]. Published reports on patients with COVID-19 have previously focused on gastrointestinal symptoms, such as vomiting, diarrhoea, anorexia, abdominal pain, and upper gastrointestinal bleeding [9], and no reports of rectal ulcer bleeding in patients with severe COVID-19 exist. To the best of our knowledge, this is the first reported case of an older patient who developed both duodenal and rectal ulcer bleeding while treating severe SARS-CoV-2 infection. Severe bleeding was controlled with systemic treatment.

The angiotensin-converting enzyme 2 receptor and transmembrane serine protease 2 (TMPRSS2) are key factors in the entry of SARS-CoV-2 into cells [10]. They are highly expressed in the gastrointestinal tract; consequently, patients with COVID-19 often exhibit gastrointestinal symptoms [11]. Notably, TMPRSS2 expression in the intestines is higher in older individuals [12, 13], suggesting that the likelihood of gastrointestinal reactions in older patients with COVID-19 is greater, and that their symptoms may be more severe. In this case report, the patient’s advanced age may have contributed to the duodenal and rectal ulcer bleeding.

**Fig. 7:**
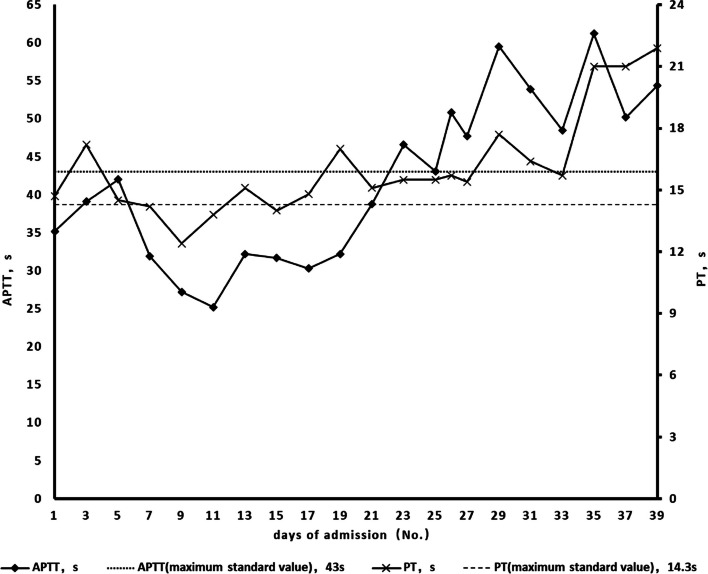
Trends in PT and APTT during hospitalisation

**Fig. 8:**
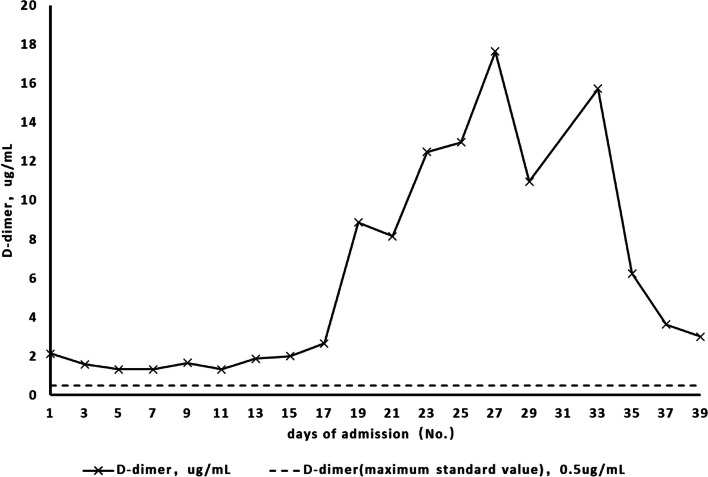
Trends in D-dimer levels during hospitalisation

Substantial evidence exists to suggest that SARS-CoV-2 can directly invade gastrointestinal tissues, resulting in the manifestation of the corresponding clinical symptoms. However, the gastrointestinal manifestations (gastric and rectal ulcer bleeding) in this case may have occurred secondary to a hypercoagulable state or cytokine activation induced by SARS-CoV-2 infection. Our patient’s duodenal and rectal ulcer bleeding symptoms mostly occurred within a few days after the nasopharyngeal swab for COVID-19 was negative. Analysis of her coagulation function during hospitalisation revealed that the patient’s coagulation function was significantly abnormal on the days before and the day of rectal ulcer bleeding. The prothrombin and activated partial thromboplastin time were higher than normal (Fig. 7), and the D-dimer levels were significantly elevated (Fig. 8). Zhang et al. previously reported that small vessel fibrin thrombi were present in 96% of ischaemic necrotic intestinal tissues resected from patients with severe COVID-19, and the D-dimer levels were significantly elevated in these patients [14]. In addition, the persistent elevation of interleukin-6 levels during the patient’s illness (Table 1) suggested that the occurrence of clinical symptoms may be related to the recruitment of macrophages [15].

Isolated rectal ulcers and malignant tumours can potentially cause rectal ulcer bleeding secondary to duodenal ulcer bleeding. However, patients with isolated rectal ulcer syndrome exhibit symptoms beyond mere ulceration, including aberrant defecation patterns, urgency, tenesmus, mucous stools, and unexplained anal pain. Furthermore, these symptoms typically correlate with complications, such as rectal prolapse and intussusception [16]. This contrasts with the clinical history obtained through patient inquiry, observed clinical symptomatology, and physical examination outcomes. Discriminating between rectal ulcers, polyps, and cancer frequently requires careful evaluation. However, the ulcers presented by this patient did not manifest features similar to the aforementioned pathologies. Notably, this patient had undergone a colonoscopy examination a year before owing to minimal rectal bleeding, and was diagnosed with internal haemorrhoids that were subsequently ameliorated through appropriate intervention. While acknowledging the pivotal role of pathological evidence, the gravity of the patient's prevailing medical condition, coupled with the ample clinical dataset at hand, informed our decision to forgo pursuing a biopsy.

Following the initial outbreak of the novel coronavirus pandemic, an increasing number of reports have suggested that the pathophysiology of this disease may be more complex than initially anticipated. In addition to its effect on the lungs, this novel coronavirus has been shown to have varying degrees of effect on multiple organ systems. This is particularly true for older adults at a higher risk of developing severe and complex complications owing to weakened immunity and complex underlying diseases. Herein, we report this unique case of an older female patient who developed duodenal and rectal ulcer bleeding during hospitalisation for severe COVID-19 pneumonia.

To our knowledge, this is the first report of an older patient who developed both duodenal and rectal ulcer bleeding while treating COVID-19-related pneumonia. This report emphasizes the need to remain vigilant about the potentially complex pathophysiological mechanisms of COVID-19 when treating novel coronavirus infections, particularly in different organ systems. Although COVID-19 may cause gastric, duodenal, and rectal ulcer bleeding, our case lacks supportive pathological evidence, and further case studies are needed to confirm this association. Overall, this case highlights a rare and severe gastrointestinal manifestation of duodenal and rectal ulcer bleeding during the treatment of COVID-19. Despite the widespread relaxation of COVID-19 restrictions, many individuals continue to be infected with SARS-CoV-2 daily. Therefore, clinicians should be aware of the diverse clinical symptoms when treating patients with COVID-19. Moreover, this case emphasises the importance of timely recognition and treatment of gastrointestinal ulcer bleeding symptoms in patients with COVID-19. As older adults are often at higher risk of gastrointestinal ulcer bleeding, increased vigilance for this symptom in this population is necessary.

## Data Availability

This case report contains clinical data from medical records in our hospital. The datasets used and/or analysed during the current study are available from the corresponding author on reasonable request.

## Abbreviations

COVID-19: Coronavirus disease 2019
ICU: Intensive care unit
SARS-CoV-2: Severe acute respiratory syndrome coronavirus
ECMO: Extracorporeal membrane oxygenation
TMPRSS2: Transmembrane serine protease 2
